# Extra-binomial variation approach for analysis of pooled DNA sequencing data

**DOI:** 10.1093/bioinformatics/bts553

**Published:** 2012-09-12

**Authors:** Xin Yang, John A. Todd, David Clayton, Chris Wallace

**Affiliations:** Juvenile Diabetes Research Foundation/Wellcome Trust Diabetes and Inflammation Laboratory, Department of Medical Genetics, Cambridge Institute for Medical Research, University of Cambridge, Wellcome Trust/MRC Building, Addenbrooke’s Hospital, Cambridge CB2 0XY, UK

## Abstract

**Motivation**: The invention of next-generation sequencing technology has made it possible to study the rare variants that are more likely to pinpoint causal disease genes. To make such experiments financially viable, DNA samples from several subjects are often pooled before sequencing. This induces large between-pool variation which, together with other sources of experimental error, creates over-dispersed data. Statistical analysis of pooled sequencing data needs to appropriately model this additional variance to avoid inflating the false-positive rate.

**Results**: We propose a new statistical method based on an extra-binomial model to address the over-dispersion and apply it to pooled case-control data. We demonstrate that our model provides a better fit to the data than either a standard binomial model or a traditional extra-binomial model proposed by Williams and can analyse both rare and common variants with lower or more variable pool depths compared to the other methods.

**Availability**: Package ‘extraBinomial’ is on http://cran.r-project.org/

**Contact**: chris.wallace@cimr.cam.ac.uk

**Supplementary information:** Supplementary data are available at *Bioinformatics* Online.

## 1 INTRODUCTION

To date, numerous common genetic variants associated with common disease [e.g. Type 1 diabetes (T1D)] have been successfully discovered by genome-wide association studies (Barrett *et al.*, 2009; Cooper *et al.*, 2008; Smyth *et al.*, 2006; Wellcome Trust Case Control Consortium, 2007). However, linkage disequilibrium (LD) means these results may only be used to identify an associated region, which usually encompasses several genes. To pinpoint the exact causal genes, under the assumption that there may be multiple variants of common and low frequency which alter disease risk, attention has turned to low-frequency variants that are unlikely to be in LD with other variants. Associated low-frequency variants are considered more likely to be causal. For example, several independent rare risk variants with implied functional roles were recently associated with inflammatory bowel disease using next-generation sequencing of pooled samples (Rivas *et al.*, 2011), a design similar to that of Nejentsev *et al.* (2009) who undertook the first such study and whose data we re-examine here.

Nejentsev *et al.* (2009) re-sequenced 144 target regions covering exons and splice sites of 10 T1D candidate genes that were previously found to be associated with T1D or related diseases. The study used 454 sequencing of 20 pools of DNA from 480 patients and 480 healthy controls (48 samples per pool). Four rare variants, single-nucleotide polymorphisms (SNPs) located in the *interferon induced with helicase C domain 1* (*IFIH1*) and the *C-type lectin domain family 16, member A* (*CLEC16A*) genes, displayed different frequencies in cases and controls according to Fisher’s exact test, and evidence for association of two of these variants within the gene *IFIH1* was replicated in independent samples. This directly implicates *IFIH1* as causal in T1D.

**Fig. 1.**
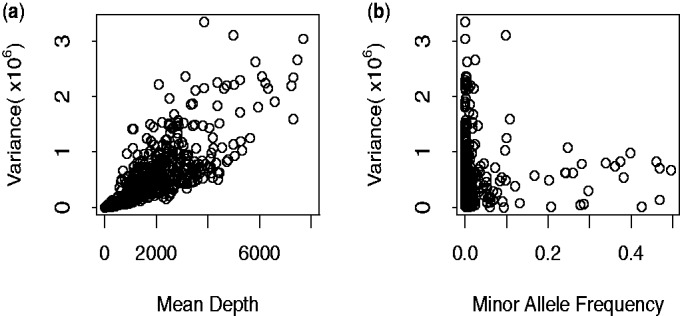
Variance analysis of the SNPs detected in Nejentsev *et al.*’s article. (**a**) Variance–mean depth comparison. Variance of depth increases with mean depths. (**b**) Variance of depth can be extreme, particularly for low minor allele frequency

A major concern with this study is that huge variation exists between pool depths and the variation is especially large for rare SNPs (Fig. 1). The variation is derived from both the pooling and the sequencing, and this is not accounted for by Fisher’s exact test which assumes a binomial variance within pools and neglects between-pool variation.

The Poisson model has also been employed for sequence count data as well as analysis of differential gene expression (Marioni *et al.*, 2008). With only one parameter, the Poisson distribution assumes the variance of reads is equal to its mean. However, the variance may exceed the mean, requiring an over-dispersed model as in our data. A negative binomial model has been used as an alternative to model the larger variance, for example in testing differential expression for digital gene expression data (Robinson and Smyth, 2007), given it has one more parameter than the Poisson distribution. The addition of this parameter, however, means the negative binomial model requires a large number of replicates to estimate the two parameters properly.

Another fully mathematically specified over-dispersed model is the beta-binomial model, which, applied to variants derived from pooled case-control sequence data, would assume a binomial distribution within pools and that the expected allele frequency of each pool is beta-distributed. Crowder (1978) used this model and calculated maximized log-likelihoods to analyse the effects from seeds and extracts on germination. Other applications of this model include the analysis of false discovery rates (FDRs) in microarray data to model the number of false rejections, yielding a less biased FDR estimator than the empirical estimator following a binomial distribution (Hunt *et al.*, 2009). The advantage of this approach is obvious: meaningful parameters and a computable likelihood which could be maximized by standard methods to calculate the relating parameters. However, maximizing a beta-binomial likelihood can be computationally difficult.

An alternative is to use a model in which only the first two moments are defined. We propose to use and extend such an extra-binomial model originally described by Williams (1982). The extra-binomial model still assumes the allele counts obey a binomial distribution within pools and also takes the between-pool variance into consideration. In this article, we compare the performance of statistical tests assuming simple binomial variation, Williams’ extra-binomial variation and our modified extra-binomial model using quantile–quantile (Q–Q) plots. We also extend our study beyond the original minor allele frequency (MAF) range used by Nejentsev *et al.* (1–3%) to include all observed single-nucleotide variation.

## 2 METHODS

### 2.1 Experimental data pre-processing

The experimental data used for the following analysis were drawn from the article by Nejentsev *et al.* (2009). The sequencing data were processed using existing software or R (R Development Core Team, 2010) as described below. BWA-SW (Li and Durbin, 2010) was used to align query 454 sequences to the NCBI36 human assembly, yielding sam files which were processed by SAMtools (Li *et al.*, 2009) to remove duplicate reads. SNPs were identified and counted by VarScan (Koboldt *et al.*, 2009), filtered by minimum base quality (≥25) and minimum total read depth (≥8).

In the absence of a variant, errors should randomly distribute across multiple pools while for a genuine rare variant excess reads of the alternative allele are expected to concentrate in one or several pools. Thus, to distinguish the errors from the true SNPs, we first excluded the common variants with MAF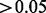 and then compared the observed pool read distribution for each remaining rare SNP to that expected under a null hypothesis of random errors only (Bansal, 2010). We assumed variants to be true rare SNPs if (i) they were not consistent with a random error model with a significant *P*-value less than 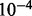 (Bonferroni correction with a prior significance level equal to 0.05) and (ii) there was at least one pool with more than two reads for the minor alleles.

For the binomial model only, SNPs and pools were further filtered by chromosome coverage—only pools with more than 80% probability to cover (include at least one read from) at least 80 out of 96 chromosomes were used. This reduced the variation between different pools by dropping pools with poor PCR or sequencing quality. SNPs were dropped if there were no valid pools for one group (case or control) at a position.

### 2.2 Statistical models

Our aim is to identify the SNPs that are associated with T1D status. We define an observation as a success if it corresponds to the major allele, otherwise it is a failure. Let 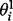 and 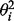 denote the expected major allele frequency for SNP *i* in control and case chromosomes, respectively. Then our hypothesis is written as
(1)
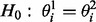

(2)
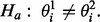

In the following models, we assume there is no systematic error in the sequencing.

#### 2.2.1 Binomial variation

For significance testing, Nejentsev *et al.* (2009) assumed a simple binomial model within pools, estimated overall allele frequencies by treating each pool equally and used these estimated allele frequencies to estimate chromosome counts of each allele. Allele counts were compared in case and control groups in a 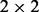 table at each SNP by Fisher’s exact test (see Section A of the Supplementary Material).

#### 2.2.2 Williams’ extra-binomial variation model (EB1)

We can relax the assumptions above by considering an over-dispersed binomial model. Here, we applied an extra-binomial model proposed by Williams (1982) to our data. With the assumption that the variation within each pool is binomial unchanged, we introduce a continuous variable 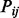 as the allele frequency of SNP *i* in pool *j* to reflect the discrepancy between different pools in allele frequency. 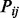 is independently distributed on (0,1) and its first two moments are defined as
(3)
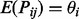

(4)
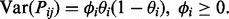



As shown below, the variance is over-dispersed when 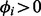. Conditionally, in pool *j* when 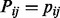, the number of reads of the major allele 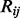 follow Bin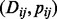 where 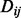 is the depth of the *j*-th pool at the *i*-th SNP and 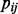 is estimated by 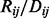. Unconditionally, deriving from Equation (4), the variance of 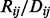 can be written as

(5)
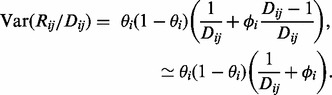

Thus, the extra-binomial model allows for between-pool variation by scaling the variance by a heterogeneity factor, 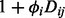, which increases with read depth. We could choose a specific distribution for 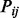 such as the beta-distribution which could induce 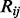 to follow a beta-binomial distribution (Zhou *et al.*, 2011). Instead, we adopt a quasi-likelihood approach and follow Williams’ method to yield an estimator of allele frequency, 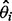, by first fitting it into a logistic linear model and then maximizing the quasi-likelihood by weighted least square iteration (see Section B of the Supplementary Material). The weighted sum of squares of residuals yields the goodness of fit statistic
(6)
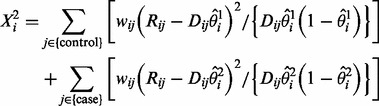

where the weight 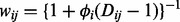. To test the significance of 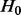, we tested the increase in 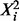 when deleting the case-control factor as suggested by Williams. This is distributed as a 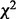 variable with one degree of freedom under the null hypothesis of no association.

#### 2.2.3 Modified extra-binomial model (EB2)

In Williams’ extra-binomial model, the parameter 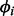 is specific to each SNP, which makes it difficult to estimate 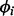 accurately given only 20 data points for each SNP. In contrast, we have many SNPs. We adopted two universal parameters *a* and *b* here instead of 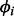 to scale the variance, such that
(7)
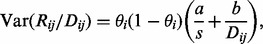

where *s* is the number of distinct chromosomes sampled in one pool, namely, 96 in this study. Ideally with no other over-dispersion, as 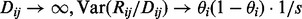. However, sequencing errors and additional variation described earlier mean 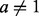. We write
(8)
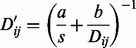

and by comparing Equation (7) with a standard binomial variance function, we see that 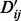 may be interpreted as the adjusted depth of pool *j* for SNP *i*.

Considering the quantities of this model,
(9)
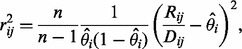

where *n* is the number of pools, namely, 20 in this study, should have expectation [see Equation (5) in the Supplementary Material]:
(10)
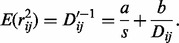

We therefore estimate the parameters *a* and *b* by linear regression of 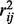 on 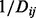, yielding 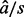 as the intercept and 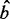 as the slope.

Given the interpretation of 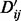 as an adjusted depth, the allele counts in pool *j* were estimated as 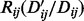 and 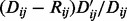. These were summed over case and control pools to form a 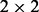 table on which a 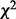 test was performed to test the significance of 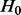.

#### 2.2.4 Accounting for sequencing error

To examine the potential influence of sequencing errors on the models, a base-specific error rate,
(11)
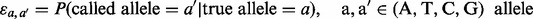

where allele *a* is the reference allele and allele 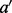 is the alternative allele was estimated by summing allele counts over all positions in our target sequence that were either not called as SNPs by VarScan or failed subsequent filtering.

We used these estimated error rates which were of the order 1–10 errors per 100 000 bases (see Supplementary Table S1) to adjust read counts of the major allele and fitted all models to the adjusted read counts
(12)
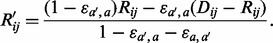



### 2.3 Simulation studies

To further evaluate the statistical performance of our models, a series of simulation studies were carried out and both Type 1 error and power under various situations were examined. The sample size was set as 500 cases and 500 controls divided into 20 pools of 50 people. The allele counts of SNP *i* in pool *j* per person were assumed to follow a gamma distribution with shape 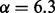 and scale 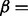 average coverage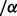 (Sarin *et al.*, 2008), where the average coverage per person was set at 40×.

We simulated three sets of 700 SNPs in each dataset (i.e. 2100 in total). The first was a set of ‘null’ SNPs, with MAF = 0; the second was a set of ‘neutral’ SNPs, with MAF in controls ranging from very rare (MAF = 0.005) to common (MAF = 0.5) and equal MAF in cases; the third was a set of ‘disease’ SNPs, with MAF in controls again ranging from 0.005 to 0.5 and in cases determined according to the control MAF and assuming a multiplicative genetic with an allele relative risk (rr) set at 1.5. As we specify our model with rr, we do not need to define the population disease rate. However, for a rare disease such as T1D, the rr is approximately equal to the odds ratio. A range of symmetric error rates (the chance of calling the alternative allele in error) from 0 to 5% was considered and applied to simulated data assuming a binomial distribution. We blinded the analysis to which SNPs were truly ‘null’ and applied the same analysis as described above for the 454 data to identify false SNPs. From these we estimated the error rates and corrected the read counts of the remaining SNPs according to Equation (12).

We performed a total of 1000 simulations in this manner, and Type 1 error rates and power were estimated from the results for the ‘neutral’ and ‘disease’ SNPs, respectively.

## 3 RESULTS

### 3.1 Simulation studies

The first two moments of our simulated data were examined, resulting in a mean sequence depth of around 1950 reads per pool and variance of 12 026.

**Fig. 2.**
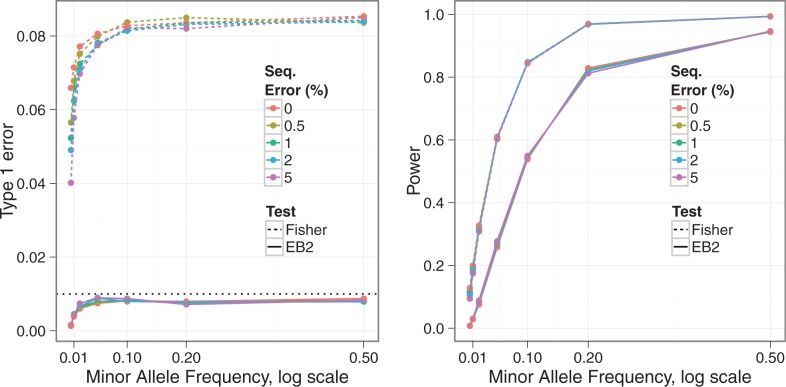
Type 1 error rate (left) and power (right) for Fisher’s exact test (Fisher) and our proposed EB2 model with the significance level 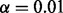. The allelic relative risk is set at rr = 1.5 for the power calculation. Sequencing error rates vary from 0% to 5%

Figure 2 shows estimated Type 1 error rates and power from our simulated data at a significance level 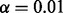. Overall, the EB2 model maintains good control of Type 1 error, across allele frequencies and error rates although tends to be conservative for rare SNPs (MAF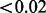). On the other hand, under Fisher’s exact test, nominal Type 1 error rates may be exceeded by a factor of nine. Note that Fisher’s test is anti-conservative in this situation, in contrast with the typical behaviour of non-parametric tests. We found the expected conservative behaviour when we simulated under a simple binomial model (data not shown), so the anti-conservative behaviour shown here appears to result from the over-dispersion. We can also see that the Type 1 error rate for Fisher’s but not EB2 is affected by the sequencing error rate. This is likely because our error correction method does a good job of correcting the simple error on average, but at the expense of increasing the noise, which is correctly dealt with by EB2 but not Fisher’s test. Power appears slightly lower for EB2 compared to Fisher’s, but not substantially so, given the difference in Type 1 error rate control. Some differentiation in power can be seen for our EB2 model with changes in sequencing error rates, with higher error rates having slightly lower power. However, the effect of sequencing error, once corrected, seems minimal given the relatively high coverage depth simulated.

### 3.2 Experimental data

After initial filtering, 473 SNPs were selected for analysis. Our estimated error rates were of the order of one error per 100 000 bases (see Supplementary Table S1), consistent with previous observations (Huse *et al.*, 2007), and had a negligible impact on association results; only the results without error correction are shown.

**Fig. 3.**
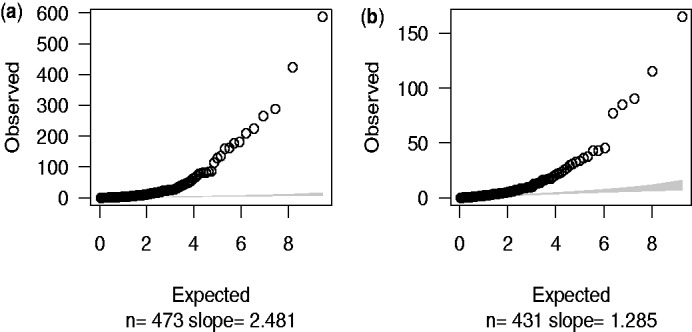
Fisher’s exact test statistics. The observed 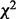 values were calculated by using Fisher’s *P*-value to calculate quantiles of 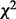 distribution. Expected values were random quantiles in 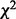 distribution with df = 1. In this and subsequent quantile–quantile plots, the shaded region is the 95% concentration band. (**a**) 473 SNPs after initial filtering. (**b**) 431 SNPs after further filtering by chromosome coverage

Although we expect many SNPs will be associated with T1D, given their location in established T1D-associated regions, we still expect them to show only limited departure from the null hypothesis due to the sample size available (even for genuinely associated SNPs, odds ratios for T1D are in the range 1–2). In contrast, a Q–Q plot of the Fisher’s exact test results (Fig. 3a) shows a large slope (slope = 2.481) and observed maximum 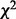 value, 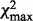, around 600 suggesting that the simple binomial model does not provide a good fit to the data. Although the slope decreases to 1.285 and 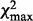 to about 150 (Fig. 3b) by filtering pools and SNPs to include only those with higher coverage (*n* = 431), this improvement was achieved at the cost of discarding data—only pools and SNPs passing the filters were counted in the test.

**Fig. 4.**
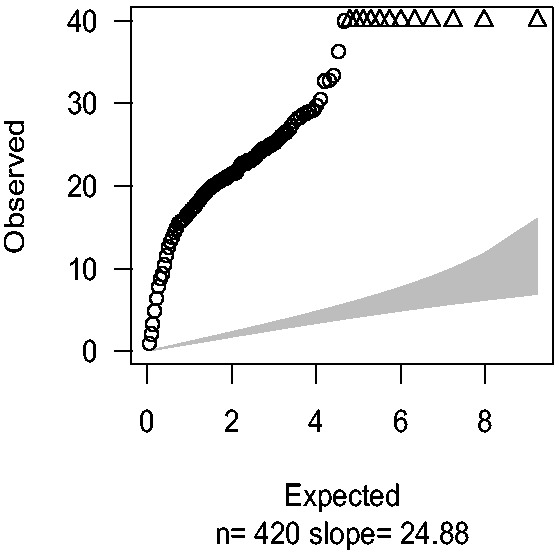
Q–Q plots were drawn by plotting observed 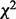 values against expected quantiles in 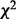 distribution with df = 1 based on Williams’ extra-binomial model (EB1). The triangles in the charts stand for the SNPs with extremely large 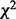 values beyond the boundary shown in vertical axis

Figure 4 shows the Q–Q plot result based on Williams’ extra-binomial model. Despite most observed 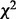 values dropping below 30, the slope remained large (slope = 24.88). Note that because variance of reads is required to estimate the over-dispersion parameter 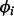, SNPs with total minor allele counts equal to zero in either cases or controls were excluded, leaving 420 SNPs to test.

**Fig. 5.**
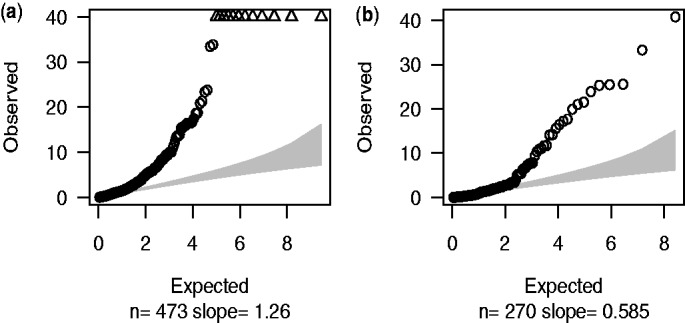
Q–Q plots of the results using EB2 model. (**a**) 473 SNPs after initial filtering were employed in the test with estimated over-dispersion parameters *a* = 0.40, *b* = 13.66. (**b**) 270 db SNPs within our target regions; *a* = 0.59, *b* = 1.27

The Q–Q plot in Figure 5a illustrates that EB2 model suits our data better with slope equal to 1.26, but the maximum sample quantile, although lower than the EB1 model, is still above 100. We recognize that despite filtering and improved models, sequencing data are still noisy and a proportion of SNPs identified may be errors. If we restrict to 270 non-novel SNPs [those in dbSNP version 128 (Sherry *et al.*, 2001)] which are less likely to be errors, all sample quantiles drop below 40 (Fig. 5b). The dominance of the EB2 model does not depend on MAF, as can be seen when SNPs are divided according to MAF (Supplementary Fig. 1).

Compared with Nejentsev *et al.*’s results, the exact values of our Fisher’s *P*-values were slightly different due to different alignment software and filters used in this article to process data. However, Nejentsev *et al.* identified four SNPs with Fisher’s *P*-values < 0.05, consistent with our results (both Fisher’s exact test and EB2 model; Table 1).

**Table 1.** Comparison of different approaches on the four SNPs identified in Nejentsev *et al.*’s article of which the first two SNPs within *IFIH1* gene were replicated in their follow-up genotyping experiment

SNP	Location	Chr.	Alleles	*P*-value			
				Nejentsev’s Fisher’s	Our Fisher’s	Our EB1	Our EB2
rs35337543	*IFIH1*, intron 8	chr2	G 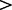 C	0.000044	0.0032	0.0029	0.016
rs35667974	*IFIH1*, exon 14	chr2	A 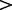 G	0.0049	0.076	0.017	0.011
ss107794688	*CLEC16A*, intron 23	chr16	C 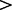 T	0.016	0.045	0.0047	0.034
ss107794687	*CLEC16A*, intron 11	chr16	C 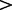 T	0.023	0.000046	0.00021	0.000005

Since different alignment software and filters were used, our Fisher’s exact results were slightly different from Nejentsev *et al.*rsquo;s. Overall, all the four SNPs still showed significance in our preferred EB2 model (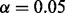).

Apart from the SNPs identified by Nejentsev *et al.* previously, there were 85 new SNPs in the EB2 model with 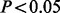. After examining the flanking 5′- and 3′-sequences (to see whether there were base repeats that are associated with erroneous SNP calls), read distribution across all pools and base qualities, seven SNPs listed in Table 2 were considered most likely to be real.

**Table 2.** The SNPs identified by EB2 model after initial filtering

SNP	Gene	Alleles	MAF		*P*-value		OR	T1D SNP	*r*^2^
			Control	Case	Fisher’s	EB2			
rs3184504	*SH2B3*	T 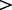 C	0.53	0.41	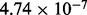	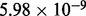	0.62	rs3184504	1
rs72650660	*CLEC16A*	C 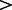 T	0.043	0.011	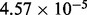	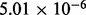	0.25	–	–
rs8052325	*CLEC16A*	A 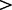 G	0.13	0.082	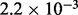	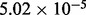	0.60	rs12708716	0.26
rs2476601	*PTPN22*	G 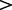 A	0.093	0.15	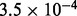	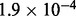	1.72	rs2476601	1
rs3827734	*PTPN22*	A 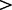 T	0.0025	0.020	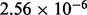	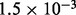	8.14	–	–
rs1800521	*AIRE*	T 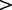 C	0.23	0.32	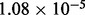	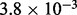	1.58	rs760426	0.04
								rs11594656	0.0029
rs28360489	*IL2RA*	C 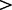 T	0.11	0.084	0.039	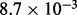	0.74	rs2104286	0.10
								rs12722495	0.62

OR is odds ratio of minor allele for T1D. *r*^2^ is the correlation coefficient with the most associated independent T1D SNPs (marker SNPs that have been identified associated with T1D before) in each region (http://www.t1dbase.org/page/Regions).

Six of these seven reside in known T1D regions, while rs1800521 (*AIRE*) is in a T1D candidate region. rs3184504 (*SH2B3*) and rs2476601 (*PTPN22*) are known T1D marker SNPs and rs28360489 (*IL2RA*) is in LD with a known T1D marker SNP (rs12722495, *r*^2 ^= 0.62) (http://www.t1dbase.org/page/Regions). Two of the remaining four, rs8052325 (*CLEC16A*) and rs1800521 (*AIRE*), are not in tight LD with known T1D SNPs (correlation coefficient 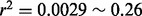), while the final two, rs72650660 (*CLEC16A*) and rs3827734 (*PTPN22*), were not found by 1000 genomes presumably due to the small MAF (Table 2), which prevents us from calculating LD.

## 4 DISCUSSION

All statistical analysis models for pooled case-control data are built on the probability distribution of allele frequency 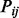 which depends upon depth (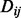), chromosome number per pool (*s*) and treatment group. Different assumptions about the distribution of 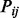 lead to different statistical tests. The binomial model used in Fisher’s exact test assumes 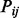 is constant across pools within the same treatment group [see Equation (2) in the Supplementary Material], while the extra-binomial variation models used in this article assume 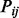 is a continuous variable that varies from pool to pool.

Note that allele frequencies estimated by sequencing pools of PCR-amplified DNA may be biased, as the PCR may preferentially amplify one allele compared to the other (Sham *et al.*, 2002). This means that allele frequencies presented in Tables 1 and 2 may deviate from the true population frequency, which can be an issue when considering the sample size required to replicate findings for rare SNPs, in particular. As this bias is likely to affect cases and controls equally when there is no underlying difference in allele frequency, it cannot be expected to inflate Type 1 error, although it may act to amplify or depress any true difference between cases and controls depending on which allele is subject to bias.

Other methods allowing for the variance in pooled sequencing have been explored. Wang *et al.* (2010) estimated allele frequencies by weighting sequencing read counts using a weight inversely proportional to the variance of the allele frequency estimate and compared these weighted estimates between cases and controls. However, no published software is available to implement this approach. An alternative approach adopted by Kim *et al.* (2010) is to estimate allele frequency by maximum likelihood and then use a likelihood ratio test to test the MAF difference between cases and controls. However, this method is affected heavily by pool size. As the authors pointed out, their approach is not applicable to pools with more than five individuals per pool. Given the main purpose of pooled sequencing is to decrease the experimental cost, a large pool size may be expected in real experimental data.

Here, instead of specifying the statistical distribution of 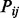, we propose a ‘quasi-likelihood’ approach which only defines the mean and variance of 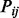. The two EB models in this article share the same mean functions of 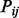 with different variance functions where Williams’ EB1 model adopted one parameter, 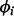, for each SNP *i* and our EB2 model adopted two universal parameters, *a* and *b*, for all SNPs.

The EB models have the advantage of including between-pool variation, compared to the standard binomial model which ignores heterogeneity of pools and thus provides a poor fit of our sequencing data (Fig. 3). However, several shortcomings are exposed in EB1: (i) the significance test is based on increases in the 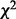 statistic, not a statistically powerful approach (McCullagh, 1983); (ii) parameter estimation is complicated requiring iteratively re-weighted least squares (see Section C of the Supplementary Material) and (iii) the small sample size (only 20 data points for each SNP *i*) means 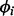 cannot be estimated precisely.

In EB2, we assumed that the parameters in the variance function are constant across all SNPs tested. By doing this, we expanded the number of data points for parameter estimation which allowed more efficient estimation and our use of GLM means it may be fitted in standard software. This could, of course, be extended to include other SNP-specific features by changing the form of Equation (8), but no other features were found to be predictive of error structure in our 454 dataset.

The improvement of EB2 over EB1 is obvious in the Q–Q plot—the slope declines to 1.26 (Fig. 4 versus Fig. 5a). Given the fact that of the 10 re-sequenced genes, 6 were in known T1D regions, we expect some SNPs would be genuinely associated with T1D and therefore expect the slope to be greater than one.

The simulation results showed that across different situations, our preferred EB2 model has an overall excellent control of Type 1 error rate. This is in contrast to Fisher’s exact test, where the observed Type 1 error rate may be as much as 9-fold the nominal rate at 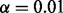.

In our 454 data, base error rates were very low. However, we included a wider range of errors in our simulations to allow us to examine the potential for our model to be applied to other technologies. Because our model only deals with increased variance, and not systematic bias induced by base calling errors, we undertake a separate error correction step which should correct the error in expectation, but may itself increase variance. This can be seen in the dependence of Type 1 error rate on the sequencing error for Fisher’s exact test, whereas our model appears to have a good control of Type 1 error rates across the range of sequencing errors considered, despite both methods being applied to the same, error-corrected, data. Note that any estimates of error rates are likely to be uncertain. Our experience is that the effect of correcting for a poorly estimated error rate in simulated data depends on MAF, the magnitude of the true error and how poorly it is estimated. In the case of our 454 data, we are lucky to be dealing with low estimated error rates which are consistent with previous estimates for this technology. Correction for these estimated error rates made no substantial difference to our conclusions. However, if the estimated error rates in other applications were larger, it might be wise to conduct some sensitivity analysis, varying the estimated error rate by factors of two or more, to examine whether associations identified are robust to misspecification of error rates.

A shortage of any model used here is the assumption of binomial error within each pool, which allows a zero count for major alleles. This is in contrast to our SNP detection principle which requires at least two supporting reads at a position to call a SNP (i.e. we should model 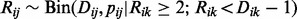 for some *k*, 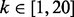). When we removed our detection criteria and instead examined all SNPs in dbSNPs version 128 (Sherry *et al.*, 2001) within our 144 target regions, the Q–Q plot showed further improvement (Fig. 5b). Additionally, a much smaller maximum sample quantile was observed in dbSNP Q–Q plot, indicating there are still errors among the full set of 473 called SNPs.

Overall, extra-binomial models appear to have better properties than the naive binomial model. They could analyse a larger range of variants with lower or more variant pool depths and our new EB2 model is more appropriate and easier to apply compared with the EB1 model proposed by Williams (1982). Work such as this is used as the basis for further confirmatory experiments and we intend to follow up the four new SNPs identified in Table 2. More accurate results lead to better targeting of these experiments and thus faster and more efficient progress to identify the causal genes in T1D.

## Supplementary Material

Supplementary Data
