# Supplementary material for: Extra-binomial variation approach for analysis of pooled DNA sequencing data
Source: Bioinformatics. 2012 Sep 12;28(22):2898–904. doi: 10.1093/bioinformatics/bts553 (PMC3496343; doi:10.1093/bioinformatics/bts553)
Supplement: Supplementary Data [file supp_28_22_2898__index.html]

Extra-binomial variation approach for analysis of pooled DNA sequencing data — Supplementary Data 

# Extra-binomial variation approach for analysis of pooled DNA sequencing data

## Supplementary Data

files

**Files in this Data Supplement:**

- Supplementary Data - pdf file
