# Supplementary material for: Extra-binomial variation approach for analysis of pooled DNA sequencing data
Source: Bioinformatics. 2012 Sep 12;28(22):2898–904. doi: 10.1093/bioinformatics/bts553 (PMC3496343; doi:10.1093/bioinformatics/bts553)
Supplement: Supplementary Data [file supp_bts553_suppl.pdf]

BIOINFORMATICS

Vol. 00 no. 00 yyyy
Pages 1–2

Supplementary Material to extra-binomial variation approach for analysis of pooled DNA sequencing data

Xin Yang [1], John A. Todd [1], David Clayton [1] and Chris Wallace [1*]

[1]Juvenile Diabetes Research Foundation/Wellcome Trust Diabetes and Inflammation Laboratory, Department of Medical Genetics, Cambridge Institute for Medical Research, University of Cambridge, Wellcome Trust/MRC Building, Addenbrooke's Hospital, Cambridge CB2 0XY, UK

Received on XXXXX; revised on XXXXX; accepted on XXXXX

Associate Editor: XXXXXXX

*to whom correspondence should be addressed

© Oxford University Press yyyy.

A BINOMIAL VARIATION MODEL

The conditional distribution of the major allele count R_{ij} in the ith SNP and the jth pool is assumed to be binomial given the observed depth D_{ij} (the total counts of both alleles for one SNP) and allele frequency (proportion of response) p_{ij}, such as

$$Pr(R_{ij} = x_{ij}|D_{ij}, p_{ij}) = \left(\begin{array}{c} D_{ij} \\ x_{ij} \end{array} \right) p_{ij}^{x_{ij}} (1 - p_{ij})^{D_{ij} - x_{ij}} \tag{1}$$

for observed allele1 counts $x_{ij} = 0, 1, \ldots, D_{ij}$. It is further assumed that the expected p_{ij} within the same treatment group is the same. Under these assumptions, for SNP i within the same treatment group,

$$\text{E}(p_{ij}) = \theta_i \tag{2}$$

$$\text{Var}(p_{ij}) = \theta_i(1 - \theta_i) \tag{3}$$

Therefore, we used $\bar{p}_i$ the arithmetic mean of $\hat{p_{ij}}$ ($\hat{p_{ij}} = x_{ij}/Dij$) to estimate θ_i. Noticeably, $\bar{p}_i$ involves no weighting of the p_{ij} by depth, which might not be appropriate when the variance of depth across pools is considerably large.

B PARAMETER ESTIMATING IN WILLIAMS' EXTRA-BINOMIAL VARIATION MODEL

Under the assumptions of this model mentioned in the main text, to estimate allele frequency θ_i, we suppose

$$\lambda_i = \ln\left(\frac{\theta_i}{1 - \theta_i}\right)$$

and λ_i fits the logistic linear model where

$$\lambda_i = \beta_0 x + \beta_1(1 - x) \qquad x = \begin{cases} 1, & \text{control} \\ 0, & \text{case} \end{cases} \tag{4}$$

The parameters involved in this model were estimated in the following iterative procedure proposed by Williams in 1982 when he programmed in GLIM (Williams, 1982) and now programmed in R in our paper.

C REGRESSION IN MODIFIED EXTRA-BINOMIAL MODEL

Equation 9 in the main document is derived in this way:

$$\begin{aligned} \text{E}(r_{ij}^2) &= \text{E}\left[\frac{n}{n-1}\frac{1}{\hat{\theta}_i(1 - \hat{\theta}_i)}\left(\frac{R_{ij}}{D_{ij}} - \hat{\theta}_i\right)^2\right] \\ &= \frac{1}{\hat{\theta}_i(1 - \hat{\theta}_i)}\text{E}\left[\frac{n}{n-1}\left(\frac{R_{ij}}{D_{ij}} - \hat{\theta}_i\right)^2\right] \\ &= \frac{1}{\theta_i(1 - \theta_i)}\text{Var}(R_{ij}/D_{ij}) \\ &= \frac{1}{\theta_i(1 - \theta_i)}\theta_i(1 - \theta_i)D_{ij}'^{-1} \\ &= D_{ij}'^{-1} = \frac{a}{s} + \frac{b}{D_{ij}} \end{aligned} \tag{5}$$

Linear regression of r_{ij}^2 on $1/D_{ij}$ for estimating the parameters a and b involved in the model was carried out using generalised linear model (GLM) by first adopting Gaussian errors to estimate initial values of a and b, and then using these to fit a GLM with gamma errors and identity link because both a and b are positive.

Since the estimated allele frequency $\hat{\theta}_i$ depends on a and b, the calculations were carried out iteratively until the difference to the previous run converged to no more than 10^{-3}.

The regression yields $a = 0.40, b = 13.66$ for all SNPs after initial filtering and $a = 0.59, b = 1.27$ for dbSNPs .

D SUPPLEMENTARY FIGURE

REFERENCES

Williams, D. (1982). Extra-binomial variation in logistic linear models. *Journal of the Royal Statistical Society. Series C (Applied Statistics)*, **31**, 144–148.

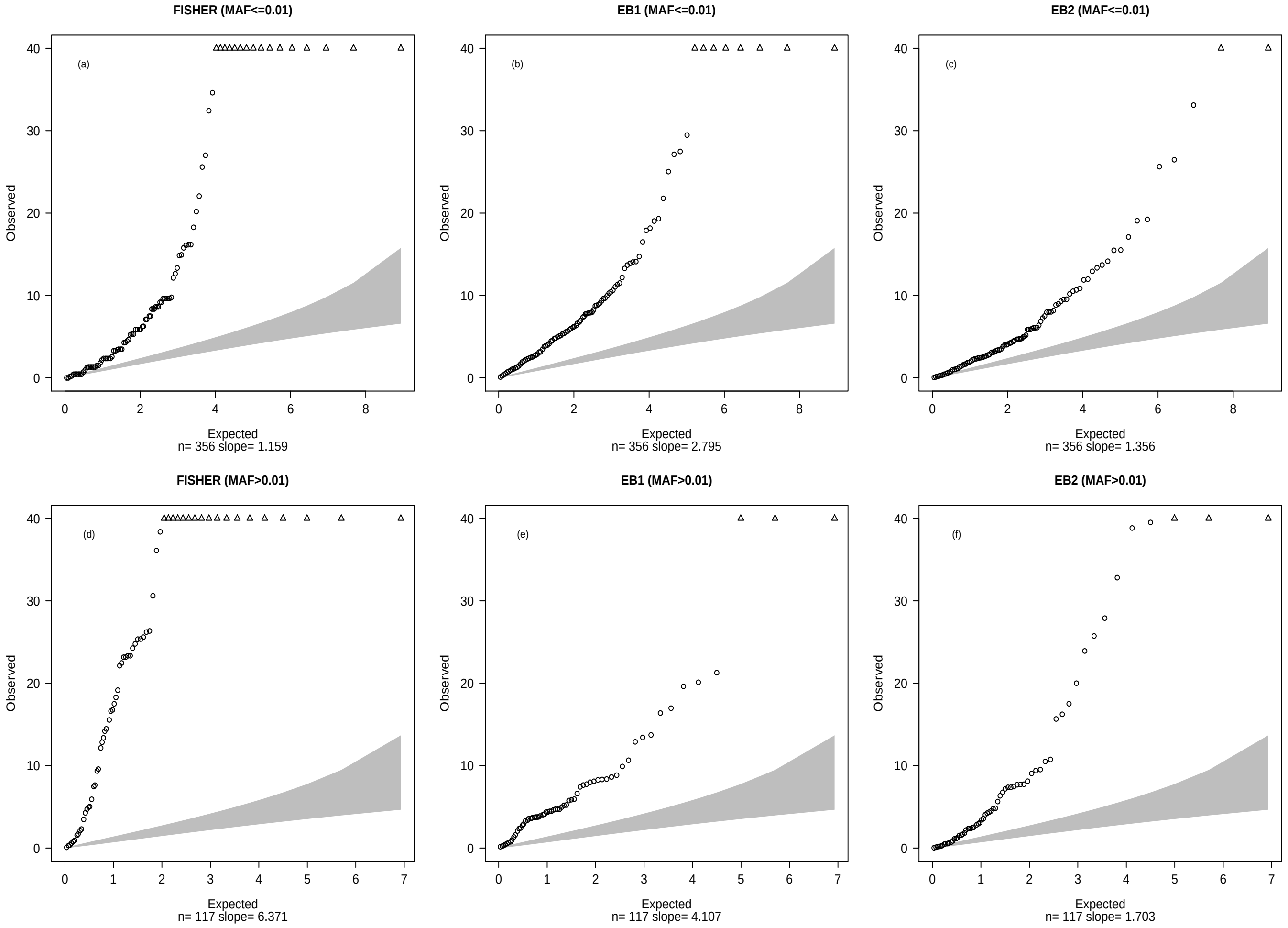

Fig. 1. SNPs were grouped according to minor allele frequencies (MAF) and three methods: Fisher's exact test, EB1 and EB2 were performed to each group. In our data, there were 356 rare SNPs with MAF less than 0.01and 117 common SNPs with MAF greater than 0.01. Q-Q plots were drawn to compare the performances of these three methods on rare and common variants.

Table 1. Sequencing error rates estimated from our experimental data (see mthod in the main text). $\varepsilon_{a,a'}$ denotes the error rate that a reference allele$a$ is miscalled as allele a'.

Error type	Error rate ($\times 10^{-5}$)
$\varepsilon_{A,T}$	2.71
$\varepsilon_{A,G}$	3.57
$\varepsilon_{A,C}$	2.36
$\varepsilon_{T,A}$	2.61
$\varepsilon_{T,G}$	2.11
$\varepsilon_{T,C}$	4.13
$\varepsilon_{G,T}$	3.70
$\varepsilon_{G,A}$	10.63
$\varepsilon_{G,C}$	1.37
$\varepsilon_{C,T}$	10.59
$\varepsilon_{C,G}$	1.16
$\varepsilon_{C,A}$	3.44
